# Listeria monocytogenes DNA Glycosylase AdlP Affects Flagellar Motility, Biofilm Formation, Virulence, and Stress Responses

**DOI:** 10.1128/AEM.00719-16

**Published:** 2016-08-15

**Authors:** Ting Zhang, Dongryeoul Bae, Chinling Wang

**Affiliations:** Department of Basic Sciences, College of Veterinary Medicine, Mississippi State University, Starkville, Mississippi, USA; University of Helsinki

## Abstract

The temperature-dependent alteration of flagellar motility gene expression is critical for the foodborne pathogen Listeria monocytogenes to respond to a changing environment. In this study, a genetic determinant, L. monocytogenes *f2365_0220* (*lmof2365_0220*), encoding a putative protein that is structurally similar to the Bacillus cereus alkyl base DNA glycosylase (AlkD), was identified. This determinant was involved in the transcriptional repression of flagellar motility genes and was named *adlP* (encoding an AlkD-like protein [AdlP]). Deletion of *adlP* activated the expression of flagellar motility genes at 37°C and disrupted the temperature-dependent inhibition of L. monocytogenes motility. The *adlP* null strains demonstrated decreased survival in murine macrophage-like RAW264.7 cells and less virulence in mice. Furthermore, the deletion of *adlP* significantly decreased biofilm formation and impaired the survival of bacteria under several stress conditions, including the presence of a DNA alkylation compound (methyl methanesulfonate), an oxidative agent (H_2_O_2_), and aminoglycoside antibiotics. Our findings strongly suggest that *adlP* may encode a bifunctional protein that transcriptionally represses the expression of flagellar motility genes and influences stress responses through its DNA glycosylase activity.

**IMPORTANCE** We discovered a novel protein that we named AlkD-like protein (AdlP). This protein affected flagellar motility, biofilm formation, and virulence. Our data suggest that AdlP may be a bifunctional protein that represses flagellar motility genes and influences stress responses through its DNA glycosylase activity.

## INTRODUCTION

Listeria monocytogenes is a Gram-positive, foodborne intracellular pathogen that causes severe illnesses in neonates, pregnant women, and immunocompromised individuals (1, 2). The switch between the pathogenic stage and the saprophytic stage of this opportunistic bacterium is accompanied by an altered transcription pattern (3), which is orchestrated by its regulatory systems, including global transcription regulators (4–9), two-component systems (10–13), and emerging noncoding RNAs (14–17).

Early studies revealed that the motility of L. monocytogenes is temperature dependent; it is nonmotile at high temperatures (37°C) and highly motile at low temperatures (28°C and below). The motility of bacteria is correlated with the temperature-dependent expression of flagella (18, 19), as well as with that of flagellar motility genes (3, 20). The expression of flagellar motility genes in L. monocytogenes is under sophisticated regulation. MogR, a transcriptional repressor, inhibits the transcription of flagellar motility genes at high temperatures (37°C and above) by binding to the promoter regions of these genes (20, 21). At low temperatures, GmaR, which is a glycosyltransferase that is capable of beta-O-GlcNAcylating its substrates (flagellin), works as an antirepressor by forming a stable complex with MogR (22), thus resulting in the nonhierarchical expression of flagellar motility genes (21). *GmaR* transcription is under the regulation of DegU (22), which is an orphan response regulator with a dispensable receiver domain that activates flagellar motility expression in a manner independent of its phosphorylation state (23–26). Remarkably, GmaR itself can work as a thermometer to regulate flagellar motility by switching the binding affinity to MogR at different temperatures (27). In addition, *sigB* can influence motility through a *sigB*-controlled *mogR* promoter (3).

Recently, a novel alkyl base DNA glycosylase (AlkD) was discovered in Bacillus cereus and has been classified as representative of a new family of DNA glycosylases, based on its unique DNA-binding protein architecture (tandem α-helical repeats, known as HEAT-like repeats). It has been shown to play a central role in *N*3- and *N*7-alkylated base repair (28–31). The objective of this study was to understand the function of the *alkD*-like gene in L. monocytogenes and determine its physiological significance with respect to biofilm formation, mobility, and stress response.

## MATERIALS AND METHODS

### Bacterial strains and culture conditions.

Listeria strains that were used in this study are listed in Table S1 in the supplemental material. L. monocytogenes strains were cultured in brain heart infusion (BHI) broth (Difco Laboratories, Detroit, MI) at 37°C. Escherichia coli DH5α was cultured in Luria-Bertani (Difco) broth. To make the deletion mutant, transformed L. monocytogenes bacteria were selected on a BHI agar plate supplemented with erythromycin (5 μg/ml) or tetracycline (10 μg/ml) when necessary. RAW 264.7 murine macrophage cells were cultured in RPMI 1640 medium (Invitrogen, Grand Island, NY) supplemented with 10% fetal bovine serum (Invitrogen) and 50 U/ml penicillin-streptomycin. All cells were cultured at 37°C in a 5% CO_2_ incubator.

For determination of the growth curve of L. monocytogenes, three strains (the F2365Δ*adlP* mutant, wild-type, and Δ*adlP*::*pMAD_adlP* mutant strains) of L. monocytogenes were cultured in BHI broth and shaken at 180 rpm for 16 h at 37°C. The cultures were then diluted 1,000-fold, and 50 μl diluted bacteria was reinoculated into 5 ml fresh BHI broth. Bacteria were incubated at 37°C in the shaker, and bacterial numbers were counted at the specified time points by plating.

### Deletion mutagenesis.

A temperature-sensitive shuttle plasmid (pMAD) was used to generate the deletion mutant (32). Using the genomic DNA of the L. monocytogenes F2365 or EGD strain as the template, the upstream and downstream regions flanking the target gene were amplified using PCR with primers (see Table S2 in the supplemental material). The PCR products were digested with the designated restriction enzymes (see Table S2) and then cloned in tandem into a pMAD_tet or pMAD_cat plasmid. The recombinant plasmid was introduced into L. monocytogenes by electroporation at 2.5 kV, 200 Ω, and 25 μF. The deletion of target genes was conducted by allelic exchange. Transformed bacteria were incubated at 43°C for 2 days for the first integration, and colonies were inoculated into antibiotic-free BHI media at 30°C and passaged three times (1 day per passage) for the second recombination. Deletion mutants were selected on BHI agar plates supplemented with erythromycin (5 μg/ml) or tetracycline (10 μg/ml) when necessary.

### RNA extraction and real-time quantitative PCR.

For real-time quantitative PCR analysis, overnight cultures of bacterial strains were diluted in fresh media and collected at the early exponential-growth phase (optical density at 600 nm [OD_600_], 0.1 to 0.2) and late exponential-growth phase (OD_600_, ∼0.8). Bacteria were pelleted, resuspended in TRIzol (Invitrogen), and disrupted in a lysing matrix B tube (MP Biomedicals, Solon, OH) by shaking in a Genie Disruptor (Scientific Industries, Inc., Bohemia, NY) for 5 min. Total RNA was extracted and treated using a RNase-free DNase kit (Qiagen, Valencia, CA) to remove genomic DNA. RNA was further purified using an RNeasy minikit (Qiagen), according to the manufacturer's instructions. Purified RNA (2 μg) was reverse transcribed into cDNA using a reverse transcription kit (Applied Biosystems, Foster City, CA). The concentration of cDNA was measured with a Nanodrop ND1000 spectrophotometer (Nanodrop Technologies, Wilmington, DE), and cDNA (approximately 100 ng) was used as the template for a 25-μl reaction system using the primers listed in Table S2 in the supplemental material. Real-time quantitative PCR was performed using a SYBR green Master kit under conditions of 95°C for 10 min and 40 cycles of 95°C for 30 s and 58°C for 60 s and an Mx3005P Real Time PCR system (Stratagene, La Jolla, CA). The internal control was *rlpM*, and the threshold cycle (ΔΔ*C_T_*) method was used to calculate relative mRNA levels.

### Motility assay.

The motility of all strains was tested on a 0.3% agar plate with 1% tryptone and 0.25% NaCl at 30°C and 37°C. Toothpicks were used to inoculate BHI broth-grown bacteria into the agar plate, and colony sizes were measured after 24 h and 48 h of incubation.

### Biofilm formation assay.

Bacteria that were cultured overnight (100 μl) were inoculated into 0.9 ml fresh BHI medium in 24-well plastic cell culture plates (Corning, NY) (∼10^7^ cells/well). After 24 h of incubation at 37°C, the unattached bacteria were removed, and fresh medium was added. After further incubations of 48 h (total 72 h), the wells were gently washed twice with phosphate-buffered saline (PBS) and then stained with 0.5% crystal violet–10% methanol. A similar incubation protocol was used to analyze biofilm formation via confocal microscopy. After 72 h of incubation, the biofilm was fixed with 3.7% formaldehyde, washed gently twice with PBS, and stained with filmTracer (Invitrogen) according to the manufacturer's protocol. *z*-axial serial collection was performed using a Zeiss laser scanning confocal microscope (LCSM, Carl Zeiss, Germany) at 2.0 μm per interval for 15 scans (*z*-axis thickness, 30 μm).

### MMS assays.

L. monocytogenes cells at the mid-exponential-growth phase were harvested by centrifugation and washed three times with PBS. Approximately 10^6^ cells were incubated with the indicated concentrations of methyl methanesulfonate (MMS) (Sigma-Aldrich Co., St. Louis, MO) for 1 h at room temperature. The numbers of recovered bacteria were counted by serial plating. We calculated the percentages of surviving bacteria by comparing the numbers of bacteria (measured in CFU) recovered from the MMS solution to the numbers of bacteria (CFU) recovered from the PBS solution.

### H_2_O_2_ sensitivity assay.

L. monocytogenes cells were prepared as described above. Bacteria were incubated with the indicated concentrations of H_2_O_2_ (Sigma-Aldrich Co.) for 10 min at room temperature, and then Micrococcus lysodeikticus catalase (Sigma-Aldrich Co.) (1,000 U/ml) was added to inactivate the remaining H_2_O_2_. We calculated the percentages of surviving bacteria by comparing the numbers of bacteria (measured in CFU) recovered from the H_2_O_2_ solution with the numbers of bacteria (CFU) recovered from the PBS solution.

### Antibiotic survival assay.

Bacterial cells (10^7^) from overnight cultures were inoculated into BHI broth supplemented with the indicated concentrations of antibiotics. After 4 h of incubation in a 37°C shaker, bacterial numbers were counted by plating. For the time-kill curve, bacteria were incubated in BHI broth supplemented with 0.5 μg/ml gentamicin and 1 μg/ml ciprofloxacin in a 37°C shaker for the indicated time periods. Bacterial numbers were counted by serial plating. We calculated the percentage of surviving bacteria by comparing the numbers of bacteria (measured in CFU) recovered after antibiotic exposure to the numbers of bacteria (CFU) at time zero.

### Macrophage survival assay.

Murine macrophage RAW 264.7 cells were seeded onto 24-well plates and incubated at 37°C in a 5% CO_2_ incubator. L. monocytogenes strains were grown in BHI medium until they reached the mid-log phase, after which they were collected by centrifugation. The bacterial pellet was washed twice with PBS and resuspended in RPMI 1640 medium. RAW 264.7 cells were infected with bacteria at a multiplicity of infection of 10:1 for 1 h. After 1 h of infection, cells were incubated with fresh medium supplemented with 100 μg/ml gentamicin to kill extracellular bacteria. Cells were washed with PBS three times at the indicated time points and then lysed with 0.2% Triton X-100. The amount of intracellular bacteria was quantified in cell lysates by serial dilution and plating.

### Infection of mice.

Four groups (4 mice per cage) of 10- to 12-week-old female BALB/c mice were injected intravenously with L. monocytogenes F2365 wild-type, F2365Δ*adlP*, EGD wild-type, or EGDΔ*adlP* bacteria at a dose of 6 × 10^3^ viable bacteria. All mice were euthanized at 72 h postinfection. The spleens and livers were collected and homogenized in PBS. All samples were serially diluted and plated on BHI agar, after which they were incubated at 37°C for 48 h. The CFU counts were recorded.

## RESULTS

### The *adlP* locus flanks LIPI-1.

**FIG 1:**
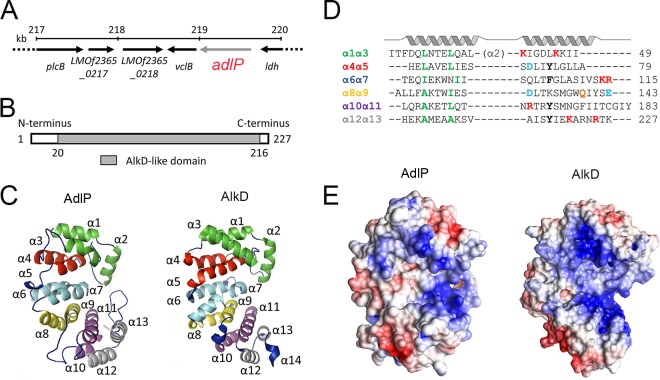
Listeria monocytogenes AdlP is structurally similar to Bacillus cereus AlkD. (A) Genomic organization of the *adlP* locus in the L. monocytogenes F2365 strain. The map was generated from the complete genome sequence of the L. monocytogenes F2365 strain. *adlP* is located between the *ldh* and *vclB* housekeeping genes and flanks the downstream region of Listeria pathogenicity island 1. (B) Domain architecture of AdlP. An AlkD-like domain is located from amino acid (aa) 20 to aa 216 (gray box). (C) Predicted three-dimensional structure of AdlP and published structure of B. cereus AlkD (protein ID 3BVS). Alpha helices are marked in order, and six pairs of anti-parallel HEAT-like repeats are shown with six different colors. (D) Structure-based sequence alignment of HEAT motifs in AdlP. Positively charged residues that contribute to the electropositive concave surface are highlighted in red. Aromatic residues are in bold, and acidic residues are in blue. A glutamine residue that has a potent role in catalytic activity is marked in orange. Conserved residues that maintain the structure of alpha helices are shown in green. (E) The solvent-accessible protein surface (with the same orientation as in the models in panel C) is colored according to electrostatic potential (red, negative; blue, positive; −5 to 5 *k*_B_*T*). Potentials were calculated with Delphi (60), and images were constructed with PyMOL (http://pymol.org/) and Chimera (http://www.cgl.ucsf.edu/chimera/).

According to the complete genome sequence data that are available for the L. monocytogenes F2365 strain, the *adlP* locus, which was previously designated “*vclA*,” is located in the downstream flanking region of the virulence cluster locus (*vcl*; also known as Listeria pathogenicity island 1 [LIPI-1]). The *adlP* locus is evolutionarily conserved among six species in the Listeria genus and is located between the *vclB* and *ldh* (lactate dehydrogenase) housekeeping genes (Fig. 1A). Along with *vclB* and *ldh*, *adlP* directly flanks LIPI-1 on the downstream side.

### AdlP is structurally similar to AlkD in B. cereus and has DNA-binding properties.

The conserved-domain search tool of the National Center for Biotechnology Information indicated that Listeria AdlP contains a putative AlkD-like domain (residues 20 to 216; Fig. 1B), which is a newly identified protein architecture in the family of DNA glycosylases. We used the SwissModel server (33) to predict the protein structure of AdlP. The B. cereus hypothetical protein (Protein Data Bank [PDB] protein identifier [ID] 1T06 A) was used as a template. A structural homology search performed with the DALI server (34) indicated that AdlP is structurally similar to AlkD (an alkyl base DNA glycosylase in B. cereus [protein ID 3BVS]), AlkF (a branched DNA-binding DNA glycosylase [protein ID 3ZBO]), and a B. cereus hypothetical protein (protein ID 1T06 A).

A cartoon representation of the Listeria AdlP homology model and the known B. cereus AlkD structure is shown in Fig. 1C. AdlP possesses a solenoid superhelical structure consisting of 13 alpha helices, which is similar to AlkD in its overall structure and protein fold. We found minor differences in the C terminus regions. AlkD has a tail of HEAT repeats, whereas AdlP has a long loop linking α12 and α13 (Fig. 1C). This loop is considered to be a unique feature of the AlkF/AlkG subfamily (35). In AdlP, except for α2, the 12 α-helices form the HEAT-like motifs: six pairs of antiparallel α-α repeats in tandem (Fig. 1C and D). A concave area on the surface of AdlP is formed by α3, α5, α7, α9, α11, and α13. Similarly to AlkD, the helices that form the concave surface are rich in lysine and arginine residues (Fig. 1D). The Delphi-calculated surface electrostatic potential showed an extremely electropositive concave AdlP surface (Fig. 1E), which is a typical feature for accommodating DNA.

### Deletion of *adlP* disrupted the temperature-dependent motility of L. monocytogenes.

To study the role of AdlP, we constructed *adlP* deletion mutants in the L. monocytogenes F2365 and EGD strains (see Table S1 in the supplemental material) by using a pMAD shuttle-vector system (32). To ensure that the deletion of *adlP* did not cause growth defects, we compared the growth kinetics of the Δ*adlP* strain, the F2365 parental strain, and the Δ*adlP*::*pMAD_adlP* strain by standard plate counting. We observed no significant differences in the growth rates of the three strains (see Fig. S1 in the supplemental material), which suggests that the deletion of *adlP* does not affect bacterial growth.

**FIG 2:**
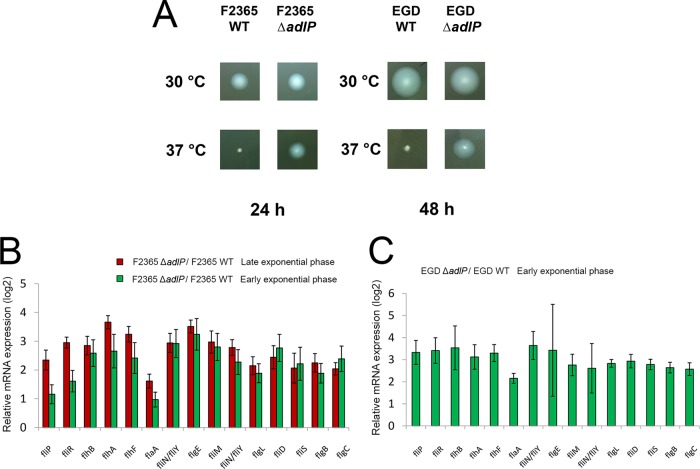
Deletion of *adlP* disrupted temperature-dependent motility and activated the expression of flagellar motility genes. (A) The motility of the strains (EGD wild-type [WT] strain, F2365 WT, EGDΔ*adlP*, and F2365Δ*adlP* strains) at 30°C and 37°C was tested using 0.3% agar plates with 1% tryptone and 0.25% NaCl at 24 h and 48 h. (B and C) F2365 wild-type and F2365Δ*adlP* strains were harvested at the early exponential-growth phase (OD_600_, 0.1 to 0.2) and late exponential-growth phase (OD_600_, ∼0.8). EGD wild-type and EGDΔ*adlP* strains were harvested at the early exponential-growth phase. Transcript levels of target genes in the F2365 (A) and EGD (B) strains were evaluated using real-time quantitative PCR. The levels of target gene transcripts in the F2365Δ*adlP* and EGDΔ*adlP* strains were normalized to the level seen with the *rplM* housekeeping gene and were then compared to those of their paternal strains. Data are means and standard deviations (SD) of results from two independent experiments with triplicates in each experiment (*n* = 6).

The motility of L. monocytogenes on soft agar is temperature dependent. We analyzed the motility of wild-type and Δ*adlP* strains on 0.3% agar with 1% tryptone and 0.25% NaCl. Consistent with previous studies, the EGD and F2365 wild-type strains were motile on soft agar when grown at 30°C but were nonmotile when grown at 37°C (Fig. 2A). Surprisingly, the F2365Δ*adlP* strain and EGDΔ*adlP* strain maintained their motility at 37°C (Fig. 2A), suggesting that the deletion of *adlP* disrupted the temperature-dependent inhibition of motility at 37°C.

### Deletion of *adlP* activated the expression of flagellar motility genes.

On the basis of our structural predictions and surface electrostatic potential calculations, AdlP had typical DNA-binding properties. Thus, we speculated that AdlP may play a role in transcriptional regulation. To address this hypothesis, real-time quantitative PCR analysis was performed to investigate the transcription levels of flagellar motility genes. RNA was isolated from strains that were harvested from the early exponential-growth phase (OD_600_, 0.1 to 0.2) and late exponential-growth phase (OD_600_, ∼0.8) at 37°C. Remarkably, the transcription levels of flagellar motility genes in the F2365Δ*adlP* strain were significantly higher than those in its parental strain in both the early and late exponential phases (Fig. 2B) at 37°C. Notably, similar results were observed in the EGDΔ*adlP* strain (Fig. 2C). These results suggest that the deletion of *adlP* activates the expression of flagellar motility genes at 37°C.

### Deletion of *adlP* impaired biofilm formation.

**FIG 3:**
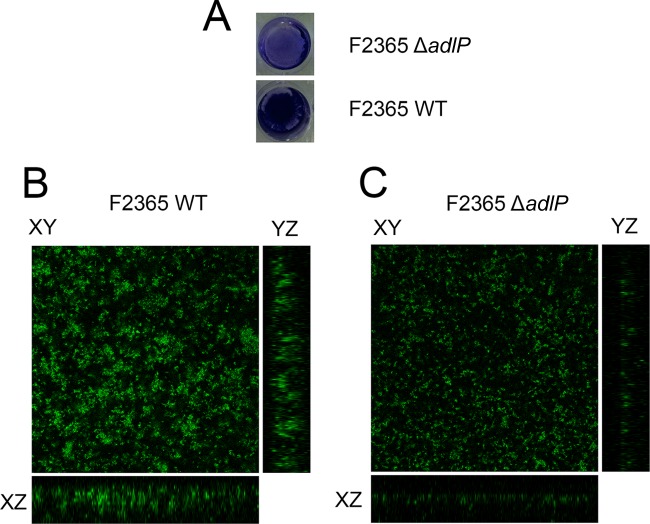
Deletion of *adlP* impaired the biofilm formation ability of the F2365 strain. The biofilm formation abilities of the F2365 wild-type (A and B) and Δ*adlP* (A and C) strains were assessed using plastic cell culture plates (A) and 8-well glass chambers (B and C). (A) The biofilm was stained with crystal violet and 10% methanol. (B and C) FilmTracer (Invitrogen) was used to stain the biofilm, and 30 μm of thickness on the *z* axis was determined by *z*-axis serial scanning using Z-stack 2-axis panels on the Zeiss confocal microscope. *z*-axis panels were constructed to determine the thickness of biofilm.

Due to the affected motility and altered expression levels of flagellar motility genes, we compared the biofilm formation characteristics of the F2365Δ*adlP* strain and its parental strain. At 3 days after incubation, the F2365Δ*adlP* strain demonstrated significantly impaired biofilm formation on plastic cell culture plates compared with its parental strain (Fig. 3A). This result was further confirmed using confocal microscopy (Fig. 3B). The thickness of the biofilm that was formed by the F2365Δ*adlP* strain was significantly less than that formed by the F2365 wild-type strain (Fig. 3B). These data demonstrate that the inactivation of *adlP* affected the biofilm formation of the L. monocytogenes F2365 strain.

### Deletion of *adlP* impaired survival in macrophages and virulence in mice.

**FIG 4:**
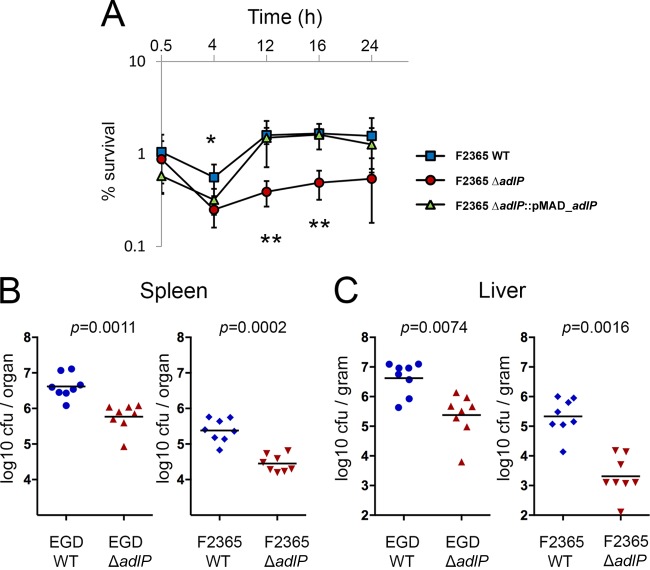
*adlP* is involved in bacterial survival in RAW 264.7 cells and is required for virulence of L. monocytogenes in mice. (A) Three strains (F2365 wild-type strain, Δ*adlP* strain, and complemented strain) of bacteria were incubated with RAW 264.7 cells at a 10:1 multiplicity of infection and allowed to replicate for the indicated time periods. Survival was calculated as the ratio of recovered bacteria to inoculated bacteria. Data are means and standard errors of the means (SEM) of the results of three independent experiments (N = 3; *, *P* < 0.05; **, *P* < 0.01) (analysis of variance [ANOVA]). (B and C) Ten- to 12-week-old BALB/c mice were infected intravenously with 6 × 10^3^ L. monocytogenes F2365 wild-type, EGD wild-type, F2365Δ*adlP*, and EGDΔ*adlP* bacteria. At 72 h postinfection, bacterial counts in the spleen (B) and liver (C) were calculated. Data are results from two independent experiments (N = 2; *n* = 8). Mann-Whitney tests were used to calculate the *P* value.

To assess the role of *adlP* in macrophage survival, we further evaluated the survival of the three strains (the F2365 wild-type, F2365Δ*adlP*, and F2365Δ*adlP*::*pMAD_adlP* strains) in RAW 264.7 cells. The Δ*adlP* strain exhibited decreased survival at all observed time points (Fig. 4A). In particular, the number of intracellular bacteria was significantly lower in the Δ*adlP* strain than in the parental strain at 12 and 16 h after infection (Fig. 4A).

To assess the role of AdlP in the infection process, we tested the virulence of the two mutants (the F2365Δ*adlP* and EGDΔ*adlP* strains) and their parental strains in BALB/c mice. The mice were injected intravascularly (i.v.) with 6 × 10^3^ viable bacteria per strain. The bacterial numbers of the F2365Δ*adlP* and EGDΔ*adlP* strains were significantly lower than those of their parental strains in the liver and spleen at 72 h postinfection (Fig. 4B and C). Interestingly, the deletion of *adlP* from the F2365 strain resulted in CFU counts in liver of infected mice that were about 2 log lower than those seen with the F2365 wild type, whereas a 1 log difference was observed for the EGD strain (Fig. 4B and C).

### The F2365Δ*adlP* strain was more sensitive to MMS and H_2_O_2_.

**FIG 5:**
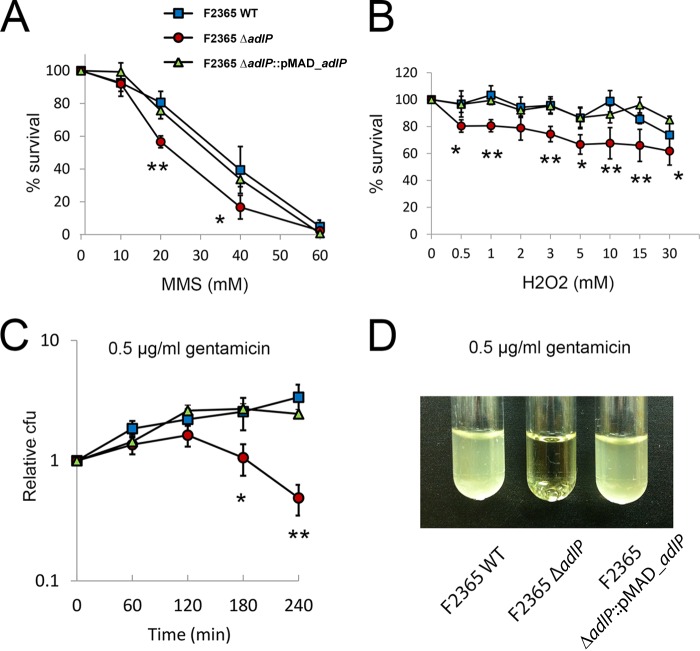
Role of AdlP in the stress response. (A) AdlP is associated with resistance to DNA-alkylating compounds. Three strains (the F2365 wild-type, Δ*adlP*, and Δ*adlP*::*pMAD_adlP* strains) of L. monocytogenes were incubated with 10, 20, 40, and 60 mM MMS or PBS at room temperature for 1 h, after which survival rates were measured. The survival percentages were calculated by comparing the bacteria (measured in CFU) recovered from MMS with the bacteria (in CFU) recovered from PBS. Data are means and SEM of the results of at least three independent experiments. (N ≥ 3; *, *P* < 0.05; **, *P* < 0.01 [ANOVA]). (B) The three strains of bacteria were treated with the indicated concentrations of H_2_O_2_ or PBS at room temperature for 10 min, and the survival rates were measured. Survival percentages were calculated by comparing the bacteria (measured in CFU) recovered from H_2_O_2_ to the bacteria (CFU) recovered from PBS. Data are means and SEM of the results of three independent experiments (N = 3; *, *P* < 0.05; **, *P* < 0.01 [ANOVA]). (C and D) Contribution of AdlP to bactericidal antibiotic resistance. (C) Three strains of bacteria were exposed to 0.5 μg/ml gentamicin for the indicated time periods, and the survival rates were measured. Data are means and SEM of the results of three independent experiments (N = 3; *, *P* < 0.05; **, *P* < 0.01 [ANOVA]). (D) The three strains of bacteria were exposed to 0.5 μg/ml of gentamicin, shaken at 37°C for 4 h, and then left at room temperature for 16 h. The image is representative of the results of two independent experiments.

Due to the structural similarity between AlkD and AdlP, we wanted to address whether *adlP* deletion affects the sensitivity of L. monocytogenes to DNA-alkylating compounds. We tested the survival of L. monocytogenes in solutions with different concentrations of an alkylating reagent, MMS, for 1 h. As predicted, the Δ*adlP* strain had a significantly lower recovery rate after treatment with 20 mM and 40 mM MMS than the wild-type and F2365Δ*adlP*::*pMAD_adlP* strains (Fig. 5A). In contrast, we found no significant differences in recovery rates after treatments with MMS solutions at a low (10 mM) or high (60 mM) concentration (Fig. 5A). The low and high concentrations represented a tolerable dose and a lethal dose for the L. monocytogenes F2365 strain, respectively. These data suggest that AdlP plays a role in MMS resistance in L. monocytogenes.

To assess the antioxidant effect of AdlP, we compared the dose-dependent and time-dependent sensitivities of the three L. monocytogenes F2365 strains to the oxidative reagent, H_2_O_2_ (Fig. 5B; see also Fig. S2 in the supplemental material). After 10 min of exposure to H_2_O_2_, the rate of survival of the Δ*adlP* strain had decreased approximately 20% to 30% compared with the rates seen with the parental and F2365Δ*adlP*::*pMAD_adlP* strains (Fig. 5B). Indeed, we observed a difference in the recovery rate after exposure of the strain to a low dose (0.5 mM) of H_2_O_2_ (Fig. 5B). Surprisingly, increasing the concentration of H_2_O_2_ (to 30 mM) did not have much effect on the overall survival rates of the three strains (Fig. 5B). We also investigated the survival of the three strains in 10 mM H_2_O_2_ at multiple time points. The Δ*adlP* strain was more sensitive to H_2_O_2_ after 1 h of exposure (see Fig. S2).

### Deletion of *adlP* impaired aminoglycoside resistance.

To examine the role of AdlP in antibiotic resistance, we tested the survival of the three strains in the presence of three categories of antibiotics: β-lactam (ampicillin), aminoglycoside (gentamicin), and quinolone (ciprofloxacin). We exposed the strains to 0.5, 0.75, or 1 μg/ml gentamicin for 4 h. The survival rate of the Δ*adlP* strain was approximately 1 order of magnitude lower than those of the parental and Δ*adlP*::*pMAD_adlP* strains in 0.5 and 0.75 μg/ml gentamicin (see Fig. S4A in the supplemental material). We also performed a time-kill study using 0.5 μg/ml gentamicin. Starting from 2 h of exposure, the Δ*adlP* strain had a significantly lower recovery rate than the parental and Δ*adlP*::*pMAD_adlP* strains (Fig. 5C). After 4 h of exposure, we observed a difference in the recovery rates of the Δ*adlP* and parental strains corresponding to 1 order of magnitude (Fig. 5C). In contrast to the wild-type and Δ*adlP*::*pMAD_adlP* strains, a survival defect was observed in the Δ*adlP* strain after 4 h of shaking at 37°C and subsequent overnight incubations at room temperature in brain heart infusion (BHI) broth with 0.5 μg/ml gentamicin (Fig. 5D). We observed similar results with streptomycin, which is another drug in the aminoglycoside category (see Fig. S3 in the supplemental material).

We also tested the survival of the three L. monocytogenes strains in the presence of a quinolone (ciprofloxacin) and a β-lactam (ampicillin). No significant differences in recovery rates were observed between the Δ*adlP* strain and the other two strains following treatments with ampicillin and ciprofloxacin (see Fig. S4B and C in the supplemental material).

## DISCUSSION

In this study, we performed real-time quantitative PCR analysis and found that deletion of *adlP* activated the expression of flagellar motility genes at 37°C in both F2365 and EGD strains. This suggests that AdlP may be involved in the transcriptional repression of flagellar motility genes at 37°C. *adlP* is evolutionary conserved and is found in all species of Listeria. Therefore, the role of *adlP* in flagellar motility regulation might also be evolutionary conserved within different species of Listeria. In contrast to other types of bacteria in which flagellar motility genes are hierarchically regulated (36), inactivation of *adlP* results in evenly elevated expression levels of flagellar motility genes in L. monocytogenes. This indicates that flagellar motility genes in L. monocytogenes are nonhierarchically controlled and is consistent with findings from previous studies (3, 21). The initial speculation regarding the regulatory function of AdlP was based on its predicted DNA-binding property. Unlike the MogR repressor, which binds to AT-rich sequences of flagellin promoters by its helix-turn-helix motif and inhibits the expression of flagellar motility genes at 37°C (21, 37), AdlP possesses 13 alpha helices and forms a solenoid structure with an extremely electropositive concave. It is highly possible that AdlP can directly bind to the promoter regions of flagellar motility genes to block the initiation of transcription. This hypothesis is supported by the biochemical property of its structurally related protein, AlkF, in B. cereus, which has a unique long loop that forms a β sheet and allows AlkF to bind to branded DNA, such as holiday junctions (35). Furthermore, solenoid structures exhibit high numbers of HEAT motifs and are able to serve as a scaffold for protein-protein interactions (38). Therefore, AdlP may also work as an adaptor protein that facilitates the activity of other regulatory machinery to regulate the expression of flagellar motility genes.

The expression of flagellar motility genes is controlled at physiological temperatures (37°C) (19), because flagellin is a major target that is recognized by Toll-like receptor-5 and Nod-like receptor NLRC4 (Ipaf) to trigger the innate immune response (39–42). Indeed, downregulation of flagellin production is used by L. monocytogenes as a strategy to avoid activation of the NLRC4 inflammasome (43). In our study, deletion of *adlP* in the F2365 and EGD strains significantly attenuated virulence in the murine infection model, compared with the results seen with their wild-type strains. We speculated that disrupted regulation in flagellar genes is one of the reasons (probably an important one) for attenuation in mice. However, it is entirely possible that other factors (such as stress responses) affected by deletion of *adlP* are responsible for the attenuation in mice. Further study, such as comparing the transcription profiles of the *adlP* deletion mutant and the wild type (by transcriptome sequencing [RNA-seq]), would reveal information beyond that regarding flagellar regulation. Similar results have been observed with flagellin-hyperexpressed strains, such as *mogR* and *flgK* mutant strains (20, 44). The activation of caspase-1 through NLRC4 by uncontrolled flagellin expression might also be responsible for the reduced survival of the F2365Δ*adlP* strain in RAW264.7 cells, as similar observations on the survival of *mogR* and *flgK* mutant strains in macrophages have been documented (20, 44).

Our results show that *adlP* inactivation constitutively activated the expression of flagellar motility genes at 37°C and severely affected biofilm formation. This suggests that regulated flagellin-based motility is required for efficient biofilm formation. Indeed, for motile bacteria such as L. monocytogenes, a transition process that allows the bacteria to switch from a motile phase to a nonmotile phase is a prerequisite for the efficient biofilm formation (45). Within the biofilm of Bacillus subtilis, flagellar motility is tightly inhibited by a molecular clutch, EpsE (46, 47). In addition to regulated flagellar activity, type IV pilus-based motility has been shown to replace flagellin-based motility in the biofilm of Pseudomonas putida (48). Interestingly, the deletion of DegU and FlaA decreases biofilm formation in L. monocytogenes (49, 50), indicating that flagella are required for biofilm formation. One possible explanation for this is that flagella are required for the initial attachment stage, because nonmotile bacteria are less attachable than wild-type bacteria (51, 52). In conjunction with previous findings, our data indicate that stage-dependent regulation of flagellar activity is required for successful biofilm formation, which involves a short stage of activation for the initial attachment and a long-term inhibitory stage during biofilm formation.

The deletion of *adlP* altered the stress response of the L. monocytogenes F2365 strain to multiple stimuli. Thus, we speculate that AdlP might be working as a functional DNA glycosylase, as the base-excision repair pathway plays a central role in counteracting DNA base lesions (53, 54). Surprisingly, recent data showed that AlkF and AlkG, which are structurally similar to AdlP in B. cereus, do not exhibit DNA glycosylase activity with respect to alkylated and oxidized bases, although a weak reduction in resistance to MMS and nalidixic acid (NAL) has also been observed in AlkF and AlkG deletion mutants (35). In our study, the deletion of *adlP* in L. monocytogenes mildly decreased the resistance of bacteria to H_2_O_2_ and MMS (with a 20% to 30% difference between the recovery rate of the mutant strain and that of wild-type strain), indicating that AdlP might play a functional role in clearing alkylated and oxidative bases in L. monocytogenes. One explanation for the minor role of AdlP in helping L. monocytogenes to counteract oxidative stress is that other glycosylases might be involved. These bases include MutM, MutY, and Nth, which are responsible for clearing DNA lesions that are caused by reactive oxygen species. Examples of DNA lesions are the highly prevalent 7,8-dihydro-8-oxo-2′-deoxyguanosine (8-oxogaunine or GO) (55) and 5,6-dihydroxydihydrothymine (thymine glycol), which is a lethal lesion that blocks DNA replication and RNA synthesis in bacteria (56, 57). Thus, double- or triple-deletion mutants are necessary to clearly reveal the role of glycosylases in oxidative stress resistance. Indeed, functional redundancies and substrate overlaps are found among DNA glycosylases. A recent report on the pathogen Neisseria meningitidis showed that a network of glycosylases is involved in the repair of oxidatively damaged bases (58). Interestingly, a time-dependent, “two-mode” killing pattern was observed in the time-dependent H_2_O_2_ killing experiment, such that the *adlP* mutant strain had two sensitive time zones (at 10 min and after 60 min) and a resistance intermediate time zone (around 30 min) with respect to H_2_O_2_ killing. This “two-mode” killing by H_2_O_2_ was also observed in E. coli in a dose-dependent manner (59) and might be another indication that a network of glycosylases exists in L. monocytogenes.

In summary, we found a putative protein in L. monocytogenes that features an overall structure similar to that of AlkD and that is encoded by a genetic determinant, *adlP* (L. monocytogenes f2365_0220 [*lmof2365_0220*]). Deletion of *adlP* activated the expression of flagellar motility genes at 37°C and disrupted the temperature-dependent motility of L. monocytogenes. In addition, *adlP* null mutants showed attenuated virulence in mice, a decreased ability to form biofilm, and less resistance to several stresses. These findings suggest that *adlP* might encode a bifunctional protein that represses flagellar motility gene expression and influences stress responses. Further studies are needed to clarify the protein function of AdlP.

## Supplementary Material

Supplemental material
