# Supplementary material for: Listeria monocytogenes DNA Glycosylase AdlP Affects Flagellar Motility, Biofilm Formation, Virulence, and Stress Responses
Source: Appl Environ Microbiol. 2016 Aug 15;82(17):5144–52. doi: 10.1128/AEM.00719-16 (PMC4988193; doi:10.1128/AEM.00719-16)
Supplement: Supplemental material [file supp_82_17_5144__index.html]

Supplemental material 

# Listeria monocytogenes DNA Glycosylase AdlP Affects Flagellar Motility, Biofilm Formation, Virulence, and Stress Responses

## Supplemental material

- Supplemental file 1 -

  Growth kinetics of *L. monocytogenes* F2365 wild type and *adlP* deletion mutant (Fig. S1); role of AdlP in the oxidative stress response (Fig. S2); contributions of AdlP to streptomycin resistance (Fig. S3) and to bactericidal antibiotic resistance (Fig. S4); bacterial stains and plasmids used in this study (Table S1); primers used in this study (Table S2).

  PDF, 572K
