# Supplementary material for: Listeria monocytogenes DNA Glycosylase AdlP Affects Flagellar Motility, Biofilm Formation, Virulence, and Stress Responses
Source: Appl Environ Microbiol. 2016 Aug 15;82(17):5144–52. doi: 10.1128/AEM.00719-16 (PMC4988193; doi:10.1128/AEM.00719-16)
Supplement: Supplemental material [file AEM.00719-16_zam999117365so1.pdf]

Supplementary Figure 1

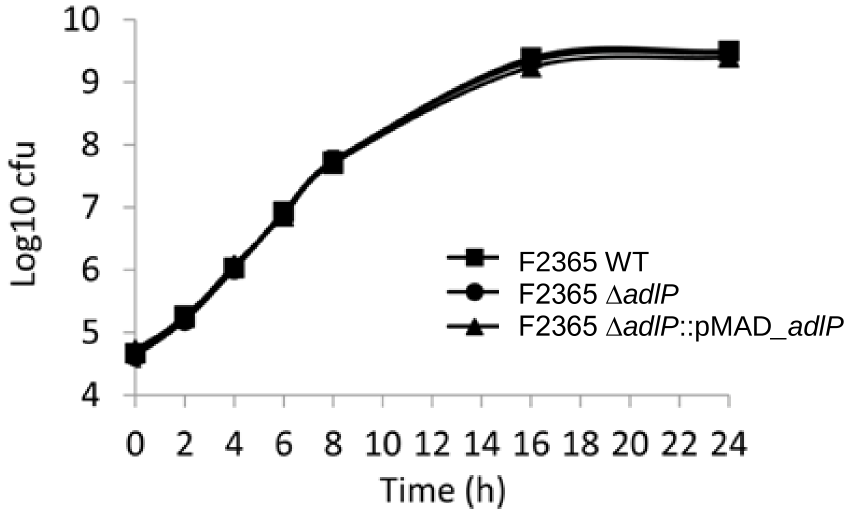

Supplementary Figure 1. Growth kinetics of *L. monocytogenes* F2365 wild type and *adlP* deletion mutant. Growth of three strains (Δ*adlP*, wild type, and Δ*adlP*::pMAD_*adlP* strains) of *L. monocytogenes* in BHI at 37°C. Data are the mean and standard error of the mean (SEM) of three independent measures.

Supplementary Figure 2

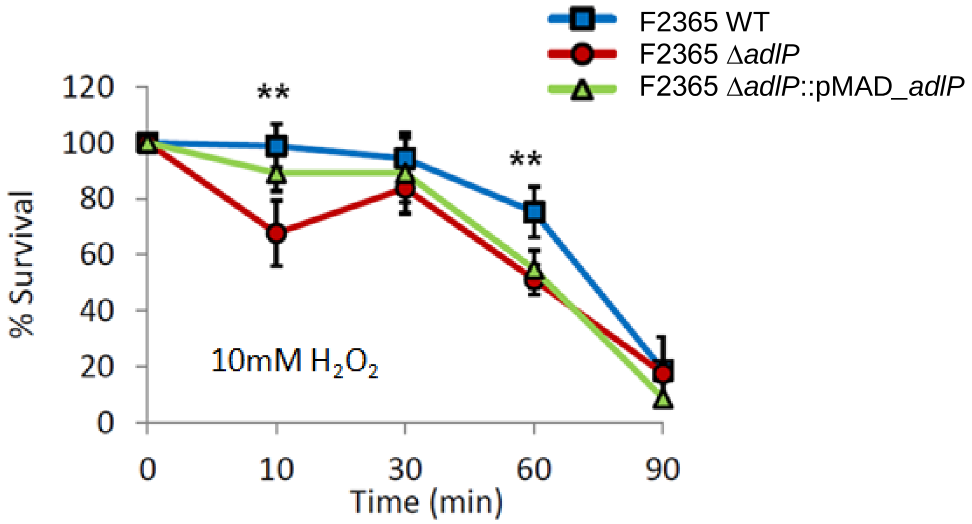

Supplementary Figure 2. Role of AdlP in the oxidative stress response. (B) Three strains of bacteria were incubated with 10mM H_2O_2 or PBS at room temperature for the indicated time and measured survival rates. Survival percentages were calculated by comparing the recovered bacteria (cfu) from H_2O_2 to the recovered bacteria (cfu) from PBS. Data are mean and SEM of three independent experiments. (N=3; * indicates $P<0.05$, $**P<0.01$, ANOVA)

Supplementary Figure 3

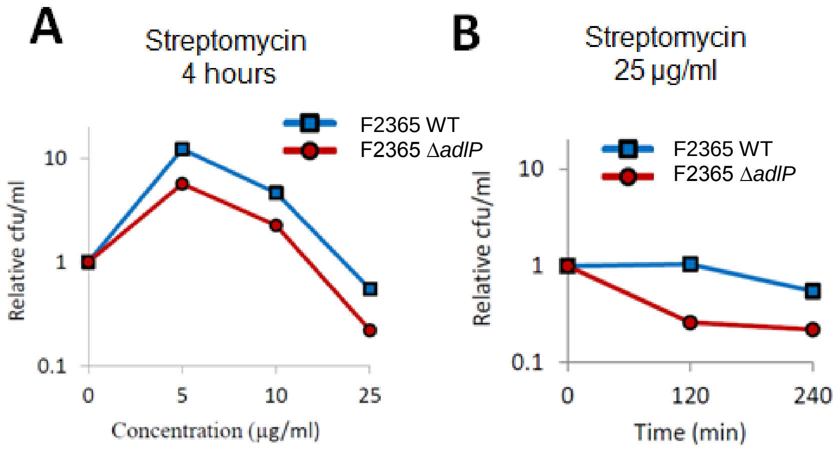

Supplementary Figure 3. Contribution of AdlP to streptomycin resistance. (A, B) Bacteria number changes following exposure to streptomycin. We exposed F2365Δ*adlP* and the parental strain to 25 µg/ml streptomycin for the indicated time (A) or to streptomycin at the indicated concentration for 4 hours (B). Data represent mean of one independent experiment of triplicates. (N=1, n=3)

Supplementary Figure 4

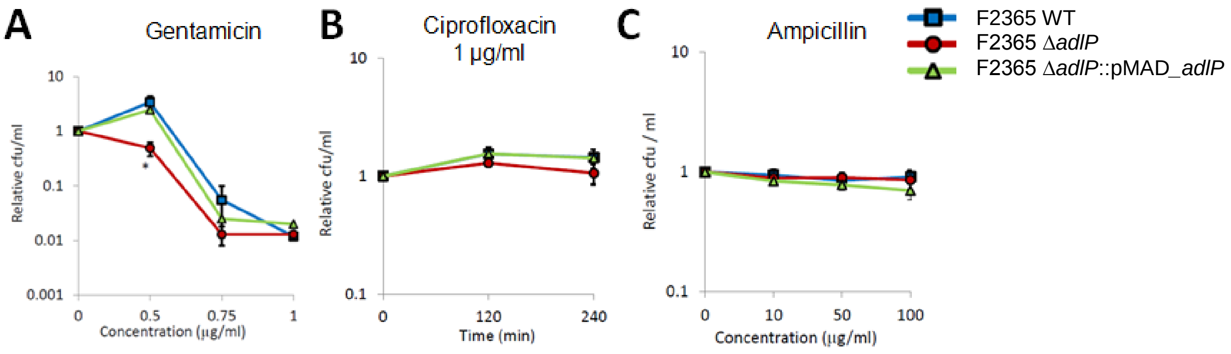

Supplementary Figure 4. Contribution of AdlP to bactericidal antibiotic resistance. (A) Bacteria number changes following exposure to gentamicin. Three strains of bacteria were exposed to gentamicin at the indicated concentration for 4 hours. Data are mean and SEM of three independent experiments. (N=3; * indicates P<0.05, **P<0.01, ANOVA) (B, C) Bacteria number changes following exposure to ciprofloxacin and ampicillin. Three strains of bacteria were exposed to 1 μg/ml ciprofloxacin for the indicated time (B) or to the indicated concentration of ampicillin for 4 hours (C). Data are mean and SEM of three independent experiments. (N=3)

Supplementary Table 1

Bacterial stains and plasmids used in this study

Bacteria strains / Plasmids	Description	Reference
**Strains**		
Listeria monocytogenes F2365	Wild type	this study
Listeria monocytogenes EGD	Wild type	this study
F2365Δ*adlP*	*LMOf2365_0220* deletion mutant, Tet^r	this study
F2365Δ*adlP* :: pMAD_*adlP*	Δ*adlP* strain harboring pMAD_F_*adlP* in genome, Tet^r, Erm^r	this study
EGDΔ*adlP*	*lmo0209* deletion mutant, Tet^r	this study
**Plasmids**		
pMAD	Shuttle vector for constructing deletion mutant , Erm^r	(Arnaud *et al.*, 2004)
pMAD_tet	pMAD vector containing Tet cassette, Tet^r, Erm^r	this study
pMAD_F_*adlP*	pMAD_tet vector containing flanking regions of *LMOf2365_0220*, Tet^r, Erm^r	this study
pMAD_E_*adlP*	pMAD_tet vector containing flanking regions of *lmo209*, Tet^r, Erm^r	this study

Supplementary Table 2

Primers used in this study

Locus/Gene name	Primer	5'-3' sequence	Restriction site
**Primers used for deletion mutagenesis**			
LMOf2365_0220	LMOf2365_0220 upstream F	GCGCGGATCCGCAGTTTGTTAGGTACCCTT	BamHI
	LMOf2365_0220 upstream R	GCGCGTCGACAAGTAGTAAGAGGTATGATA	SalI
	LMOf2365_0220 downstream F	GCGCCTCGAGGCATGCTTTTAGGATTTGAG	XhoI
	LMOf2365_0220 downstream R	GCGCAGATCTTCAGGACGTTCAACGCTTCA	BglII
lmo0209	lmo0209 upstream F	GCGCGGATCCGTCATATGCTTCGTTAGGTG	BamHI
	lmo0209 upstream R	GCGCGTCGACAGCTTGTAAAGTTCCAGATGC	SalI
	lmo0209 downstream F	GCGCCTCGAGCTTGTAGCTCGGTGTCAAGT	XhoI
	lmo0209 downstream R	GCGCAGATCTGTGGATGAAGTTATGGCAAGC	BglII
**Primers used for real-time PCR of F2365 strain**			
rplM	LMOf2365_2570F	ACGCATTTCGCCTGCAGTAC	
	LMOf2365_2570R	TCCACATATCGACACTGGAG	
fliP	LMOf2365_0712F	TGCTTGTTGGACTGGCACTG	
	LMOf2365_0712R	TCCTTCTGCTTTCAACATCATG	
fliR	LMOf2365_0714F	TCACTTCAAGGCATGGATTAC	
	LMOf2365_0714R	ATACTACCCATAATCGGGAG	
flhB	LMOf2365_0715F	ACGTTGCTTTCATACGTGGC	
	LMOf2365_0715R	CTGCGATTACAAGGAAGAGC	
flhA	LMOf2365_0716F	AATTGACGGACTTGCTGATGA	
	LMOf2365_0716R	ATGCCATCAAGATCATTCGG	
flhF	LMOf2365_0717F	ATTGTCAGCGATGCGTCTCG	
	LMOf2365_0717R	TACTTGCTTAAACTGCTTCCG	
flaA	LMOf2365_0726F	GTATGCGTCAATTAGCAGTAC	
	LMOf2365_0726R	AGTAGCAGCACCTGTAGCAG	
fliN/fliY	LMOf2365_0729F	CCAAGTAGACAATATTGGCGT	
	LMOf2365_0729R	ATTCCGAAGTTCTCGTCCATC	
flgE	LMOf2365_0733F	CGTAATGGGTTACGCAACAG	
	LMOf2365_0733R	CATATACAGAAAGCTCGGAAG	
fliM	LMOf2365_0735F	CATCCAATCTCACCAGAAATC	
	LMOf2365_0735R	CACGCCAATGCGCATTGTAC	
fliN/fliY	LMOf2365_0736F	GTTCCAGAGCCAGAACCAGT	
	LMOf2365_0736R	GACGTTCATTAAACTCGAACTC	
flgL	LMOf2365_0742F	GCTAACTCAGAAGACGATGG	
	LMOf2365_0742R	TCATCGCACTACCATCATGG	
fliD	LMOf2365_0743F	CAATGCACTAACAAGCACAATG	
	LMOf2365_0743R	AACGCCTTCTTTGTCGACACT	
fliS	LMOf2365_0744F	ACAAAGCACTCGAACAACTAC	
	LMOf2365_0744R	CAGAAATCCATTCATATAAGCC	
flgB	LMOf2365_0746F	CGAGCATCTGGTCAGAATAG	
	LMOf2365_0746R	CATTTCAGAAGTGACGTTGAC	
flgC	LMOf2365_0747F	CAATGGATGGAAGTAAGCTCC	
	LMOf2365_0747R	CGTTTGACGCTTCCTGTGTC	

Primers used for real-time PCR of EGD strain

rplM	lmo2597F	ACGCATTTCGCCTGCAGTAC
	lmo2597R	TCCACATATCGACACTGGAG
fliP	lmo0676F	TGCTTGTTGGACTGGCACTG
	lmo0676R	AACTGGATCTTCTCCTTCTGC
fliR	lmo0678F	CTTGTCGCTACAAGGAATGG
	lmo0678R	ATACTACCCATAATCGGGAG
flhB	lmo0679F	TCTTATGTGGCTTACATTGGC
	lmo0679R	AAGTCGAGCAGCCCTATTAC
flhA	lmo0680F	TTGACGGATCAGATGATAACG
	lmo0680R	ATTGCGTACGATAGCCGATG
flhF	lmo0681F	GAGTGAAGTGGCAATAGCTG
	lmo0681R	CGTCGATTGTGCTAGTTCC
flaA	lmo0690F	GTATGCGTCAATTAGCAGTAC
	lmo0690R	AGTAGCAGCACCTGTAGCAG
fliN/fliY	lmo0693F	GGACATTGCCGAGTTAAGC
	lmo0693R	ATTCCGAAGTTCTCGTCCATC
flgE	lmo0697F	CTTCCGAGCTGTCTGTATAC
	lmo0697R	TTAAGTGTCCCTGTCACTGG
fliM	lmo0699F	CATCCAATCTCACCAGAAATC
	lmo0699R	TTCATAATCTCTTCCACCGAG
fliN/fliY	lmo0700F	ATTGGCATAGGTTCAGCCTC
	lmo0700R	TTGAGCGTAGTCCTTCGACA
flgL	lmo0706F	GCTAACTCAGAAGACGATGG
	lmo0706R	CTTCTACTTCTAACGTGTCCG
fliD	lmo0707F	GTAACCAACTACATCCCAGG
	lmo0707R	GCTTGTTAGCGCGTTGTATG
fliS	lmo0708F	CAAAGCACTCGCAGAACTAC
	lmo0708R	CAGAAATCCATTCGTATAAGCC
flgB	lmo0710F	ATCACTTGGCTCGAGTTTGC
	lmo0710R	CATTTCAGAAGTGACGTTGAC
flgC	lmo0711F	CAATGGATGGAAGTAAGCTCC
	lmo0711R	CCTGTTAAAGCTGTTTCGAAC

References

Arnaud, M., A. Chastanet & M. Debarbouille, (2004) New vector for efficient allelic replacement in naturally nontransformable, low-GC-content, gram-positive bacteria. *Appl Environ Microbiol* **70**: 6887-6891.
